# Different measures of working memory decline at different rates across adult ageing and dual task costs plateau in mid-life

**DOI:** 10.1177/17470218251351307

**Published:** 2025-06-06

**Authors:** Alicia Forsberg, Clément Belletier, Agnieszka Graham, Stephen Rhodes, Pierre Barrouillet, Valérie Camos, Nelson Cowan, Moshe Naveh-Benjamin, Robert H Logie

**Affiliations:** 1 The University of Sheffield, Sheffield, UK; 2 Université Clermont Auvergne, Clermont-Ferrand, France; 3 Queen’s University Belfast, Belfast, UK; 4 Urology Institute, University Hospitals Cleveland Medical Center, Cleveland, OH, USA; 5 University of Geneva, Geneva, Switzerland; 6 University of Fribourg, Fribourg, Switzerland; 7 Department of Psychological Sciences, University of Missouri, Columbia, MO, USA; 8 The University of Edinburgh, Edinburgh, UK

**Keywords:** Working memory, cognitive ageing, processing, memory, dual-task, dual-task cost

## Abstract

Working memory allows us to store information in mind over brief time periods while engaging in other information-processing activities. As such, this system supports cognitive *dual-tasking*, that is, remembering information while performing a concurrent processing task. Age-related dual-task deficits have been proposed as a critical feature of lifespan cognitive decline. However, evidence regarding such deficits has been mixed, and knowledge of the conditions under which such deficits appear remains elusive. Moreover, several studies have suggested that different aspects of working memory decline at different rates with age and that age-related change is not necessarily linear. We explored lifespan changes in 539 participants (aged 15–90 years) on several memory, processing, and dual (combined) tasks. We addressed two research questions: (1) Does the magnitude of dual-task costs change across the lifespan? (2) Do different measures of memory, processing and dual-tasking, all decline at the same rate with age? We found that younger-young adults outperformed all other participants on dual-task measures. However, deficits did not appear to increase from the age of 35 years into older age, suggesting that dual-task ability declined in early adulthood but not thereafter between midlife and older age. Processing performance appeared to decline linearly and more rapidly with age than memory performance. Our finding that for some measures, the largest changes occurred in the transition from early to middle adulthood provides an interesting contrast to the widely held assumption that cognition declines continuously across the adult lifespan.

As people grow older, some aspects of cognitive performance tend to decline. An accurate understanding of healthy ageing is necessary to make informed diagnoses regarding cognitive health status throughout adulthood. Dual-tasking (i.e. doing two cognitive operations at the same time) is considered a huge burden of modern life, and previous research suggests that this ability may be especially impaired with ageing. However, our study showed that the ability to dual-task may remain relatively intact from the age of 35 years and onwards, at least for part of the population, despite continuous age-related changes in the ability to remember and process information separately. We also found that the ability to process information appeared to decline more rapidly than the ability to remember information. Our findings highlight the importance of considering not only aspects of cognition that are especially prone to accelerated age-related decline, but also those that are relatively intact across the adult lifespan. For example, identifying age-related strengths and weaknesses may inform age-inclusive design of websites and online applications.

Age-related memory decline has consequences for the ability to carry out everyday activities required for independent living (Tomaszewski Farias et al., 2009). Working memory is often defined as a system that allows us to keep a small amount of information in mind over brief time periods while engaging in other information-processing activities (see reviews in Logie et al., 2021). As such, the working memory system is seen as underpinning most complex cognitive activities (Conway et al., 2007), including our ability to remember and process information simultaneously (i.e. *dual-tasking*). Dual-task ability is essential in most daily cognitive activities – such as remembering your colleague’s coffee order while crossing a busy street – and has been explored extensively in young adults (e.g. Barrouillet et al., 2004, 2011; Cocchini et al., 2002; Doherty & Logie, 2016; Thalmann & Oberauer, 2017; Vergauwe et al., 2021).

There is extensive evidence that healthy older people perform more poorly than younger healthy people on a wide range of cognitive tasks. This age-related cognitive change has been demonstrated so frequently that more than 20 years ago, Perfect and Maylor (2000) argued that further demonstrations of cognitive performance differences between groups of younger and older adults add little to existing knowledge and thus are not very interesting. They referred to the expectation that older people would perform more poorly than younger people on any task you care to mention as the ‘dull hypothesis’. Moreover, cohorts of younger and older adults will have had different educational and life experiences and may differ in factors other than their chronological age. For example, older people might perform a task using a different cognitive strategy than younger people (e.g. Forsberg et al., 2019, 2020), so results from a comparison between, for example, 18 to 30 year olds and 65 to 80 year olds might not reflect only age differences and should be interpreted with caution.

One approach to avoid such cohort effects is to conduct a longitudinal study of the same individuals over several decades, and such studies can be very valuable (e.g. Deary et al., 2007; Hülür et al., 2018; P. Rabbitt et al., 2004; Small et al., 2012). Several different classification systems and labels have been proposed to characterize which types of cognitive mechanisms ‘hold’ and which ‘do not hold’ with advancing age in adulthood (see Salthouse, 2010). A distinction is often made between crystallized abilities and fluid abilities. The former refers to acquired knowledge and skills that tend to improve throughout most of the lifespan and remain stable in healthy older age. The latter refers to solving novel problems and flexible thinking that tend to decline across the adult lifespan (Baltes et al., 1999; Cattell, 1943; McArdle et al., 2002). However, as outlined by Salthouse (2010), these labels may be misleading, as the literature suggests that abilities like memory and speed, which are highly sensitive to age, appear psychometrically distinct from fluid ability. Moreover, there is evidence that participants in longitudinal studies show practice effects even when periods of several years elapse between test sessions, and the drop-out rate can lead to a diminished sample size as the study progresses (e.g. P. Rabbitt et al., 2004). Also, the cognitive assessments chosen at the start of a decades-long study may be less suitable for testing theories that develop or change over time. An alternative is to run a cross-sectional study but with a sufficiently large sample size and broad age range to allow for the possibility of detecting differences between groups of participants who are closer in age, and therefore likely to have had very similar educational and life experiences (e.g. Johnson et al., 2010; Maylor & Logie, 2010).

More interesting than the ‘dull hypothesis’ may be to study the age-related trajectory of performance on a range of different cognitive tasks, with age as a continuous variable across the adult lifespan rather than as a binary grouping variable. This would allow an exploration of whether different cognitive abilities show the same or different rates of age-related decline, whether there are cognitive abilities that do not decline across the adult lifespan, and whether or not any cognitive decline is linear across age. Pursuing these kinds of questions on cognitive ageing, some studies have shown that different aspects of working memory and short-term storage decline at different rates as we get older. Some studies of the lifespan trajectory of cognitive ability have suggested that some aspects of age-related cognitive decline begin in healthy adults in their 20s and 30s (e.g. Johnson, et al., 2010; P. Rabbitt & Goward, 1994; Salthouse, 2009; Thompson et al., 2014). For example, Thompson et al. (2014) measured age-related changes in cognitive-motor performance in adulthood using data from a complex video game and found that age-related slowing of within-game, self-initiated response times began around the age of 24 years (although, see von Krause et al., 2022). Johnson et al. (2010) and Logie et al. (2015) observed different rates of decline for different working memory abilities, performed as single tasks by over 95,000 participants from across the adult lifespan. Tests of visual short-term memory showed significant decline by age 25, compared with 20 year olds (groups with similar life and educational experiences), whereas a measure of verbal memory span showed no age-related decline across groups of participants under the age of 65 years. Other studies have shown that response time, response time variability and retention of abstract visual patterns decline with age more quickly than does verbal memory span (e.g. Anstey et al., 2005; Greene et al., 2020; Park et al., 2002; P. M. A. Rabbitt, 2000; Zuber et al., 2019). In an internet study^1^ with over 318,000 participants aged 8 to 50 years, Maylor and Logie (2010) reported that their prospective memory measure tended to decline between the ages of 20 and 50, whereas the measure of retrospective memory tended to improve or remain stable over the same age range, although both improved during childhood and adolescence.

The studies reviewed thus far have focused primarily on how the performance of a range of single tasks changes differentially across adult ageing. However, as we review in the next section, there is a lack of consistency in the previous literature regarding age-related change in the ability to perform two tasks concurrently, such as holding material in memory while performing an unrelated task. Therefore, the primary motivation for the current study was to further explore the fine-grained trajectories of age-related changes in different aspects of working memory, focusing on age-related changes in memory and processing abilities under single and dual tasking conditions.

## Age-related dual-task deficits

Evidence from early research suggests that age-related decline in short-term memory is greater when concurrent processing of an additional task (dual task) is required (e.g. Broadbent & Heron, 1962; Wingfield et al., 1988). This is supported by more recent work (Bier et al., 2017; Jaroslawska et al., 2021; Rhodes et al., 2019, 2021), but the evidence for this finding is mixed (see reviews by Kilb & Naveh-Benjamin, 2015; Logie et al., 2015; Naveh-Benjamin & Cowan, 2023). Whereas one meta-analysis suggested that older adults were considerably more impaired than younger adults when performing a complex span task (Bopp & Verhaeghen, 2005), other individual studies present conflicting results (e.g. Jenkins et al., 1999). In a meta-analysis of age-related dual task costs, Jaroslawska and Rhodes (2019; see also Wasylyshyn et al., 2011) found overall evidence for age-related decline in dual-task performance, but for studies in which the task difficulty level was adjusted (titrated) to create a common baseline across age groups, the age effect was reduced to at or near zero (e.g. A. Baddeley et al, 1986; Logie et al., 2004; Somberg & Salthouse, 1982; see review in Logie et al., 2015). Rhodes et al. (2019) found age differences in dual-task costs, even when single task demand was adjusted to the span of each participant to ensure a common baseline (see also Rhodes et al., 2021), but found no age-related decline in the ability to prioritize one or other task under dual-task conditions. Although Rhodes et al. (2019) sampled participants across the adult age range (18–81 years), and treated age as a continuous variable, the overall sample size of 164 participants resulted in modest numbers within each age decade. Moreover, Rhodes et al. (2019) used only one measure of memory, and both the memory (letter sequence recall) and processing task (arithmetic verification) involved verbal ability. Previous studies showing a lack of age-related dual task cost have used contrasting tasks such as verbal digit span and perceptuo-motor tracking (e.g. A. Baddeley et al., 1986; Della Sala et al., 2010; Logie et al., 2004; MacPherson et al., 2007). The primary aim of the study reported here was to explore the age-related trajectory of dual-task performance by using a larger sample than many previous group studies and exploring age-related change in dual-task combining a verbal memory task with a visuo-spatial processing task (rather than both being verbal), with age as a continuous rather than a binary grouping variable. We included other indices of memory and response times, which allow comparisons of trajectories of age-related differences in dual task, memory and processing ability.

In the current study, we also attempted to address some of the possible reasons for the inconsistency across previous studies regarding age-related changes in dual task performance. One approach to seeking a resolution for mixed results across studies is for the researchers associated with those contrasting results to work in collaboration within a single project, an approach known as ‘adversarial collaboration’ (Clark et al., 2022; Cowan et al., 2020; Doherty et al., 2019; Kahneman, 2003; Logie, 2023). The study reported here was conducted within a larger adversarial collaboration project ‘Working Memory Across the Adult Lifespan’ (WoMAAC – womaac.psy.ed.ac.uk) involving three groups of researchers who had developed contrasting theoretical frameworks for working memory, with different implications for age-related cognitive decline (see Jaroslawska et al., 2021; Rhodes et al., 2019, 2021). Across studies reporting divergent outcomes, we note that dual-task costs have been measured in numerous different ways (see Jaroslawska & Rhodes, 2019). Here, we compared performance when completing either one or two tasks, as well as a measure that required both memory and processing, interleaved. Our primary measure was conceptually similar to a Brown-Peterson task and may be described either as a working memory or a short-term memory task (see Cowan, 2017). This specific task was chosen due to similarities with recent, related, dual-task studies, which were designed using an adversarial collaboration approach (Rhodes et al., 2019, 2021).

## Do memory, processing and dual-task abilities decline at the same rate with age?

Given the previous evidence that different aspects of working memory decline at different rates across the adult lifespan (e.g. Johnson et al., 2010; Park et al., 2002; P. M. A. Rabbitt, 2000; Salthouse, 2015), a secondary aim of the current study was to explore the relative rates of age-related change in memory and processing abilities as single tasks and when performed concurrently (dual task). With this approach, we sought to move beyond simply contrasting the overall scores of younger and older adults on some cognitive tasks by exploring differences in the rate of decline in different aspects of cognitive function, including dual task across participants from young adulthood through early and late middle age to more senior years. Identifying aspects of performance, and specifically dual tasking, that show more or less pronounced age-related decline, might help us understand which aspects of working memory performance, and cognition more broadly, decline with age and the rate at which any such decline occurs. It also has important implications for designing a society that is accessible across the lifespan, for instance, by creating online environments that rely on abilities that are relatively intact in older people while providing support for abilities that are impaired with age.

In summary, we explored the lifespan trajectory of memory, processing, and dual-tasking ability, to address two questions (1) Does the magnitude of dual-task costs change across the lifespan? (2) Do different measures of memory, processing and dual-tasking, all decline at the same rate with age? More specifically, we explored the lifespan changes in memory, processing, and dual-task ability in 539 participants, performing seven tasks assumed to rely on memory and/or processing ability.

## Method

### Participants

Participants were recruited to perform the task in our lab or remotely, online. The in-lab participants were recruited in Edinburgh, UK, using the local participant volunteer panel. The online study was advertised through the project website, social media, flyers and public research talks. Additionally, we shared the link to the study with colleagues, acquaintances, students and local interest organizations, and encouraged them to share the link within their networks. Given the anonymity of the data, we cannot report the number of online participants who were recruited through specific methods. A total of 541 participants completed all the tasks, either online at a location and time of the participant’s choice, and with no experimenter supervision, or online in the lab with an experimenter present to answer questions and ensure a quiet environment for the duration of the testing. The lab-based testing was carried out as a check on the integrity of the unsupervised data collection. We excluded one participant for not entering their age and one 19-year-old participant for unrealistically fast average response times in the simple Response Time (RT) task (16.25 ms), achievable by pressing the response key continuously throughout the task. No other participants had missing data or values that indicated cheating. Of the final sample (*N* = 539 participants; 77.8% Female, 21.2% Male and 1.1% who ‘preferred not to say’), 445 completed the study in their chosen environment, and 94 completed the study in the lab. Participants in their own environment were able to opt in to a raffle to win a voucher for their participation, and in-lab participants received a small cash payment. Participants ranged from 15 to 90 years old (age *M* = 48.0, *SD* = 21.1 years). A preliminary analysis indicated that patterns of data were very similar from unsupervised participants and from those who participated under experimenter supervision in the lab.^2^ For the analyses presented in this paper, we combined data from online and in-lab participants. The sample size was determined based on the availability of participants, as the study was available online to all interested participants who came across it. See Table 1 for demographic information by age group. See Supplemental Material for more detailed demographics, including participant country and level of education. While the current study included participants from 35 unique countries, data on race and ethnicity were not collected, which limits generalizability (see Roberts et al., 2020). Data were collected between May 2019 and October 2020.

**Table 1.** Participant demographics by age group (*N* = 539).

Age range	*N*	Age, *M* (*SD*)	Female/male/prefer not to say (*N*)	In-lab (*N*)	Unique countries (*N*)	English speaker from birth
15–24	132	20.5 (2.3)	105/25/2 (79.5% F)	59 (44.7%)	19	84 (63.6%)
25–34	64	28.8 (2.5)	45/17/2 (70.3% F)	9 (14.1%)	20	31 (48.4%)
35–44	37	39.4 (2.5)	29/7/1 (78.4% F)	6 (16.2%)	9	18 (48.6%)
45–54	49	50.5 (2.9)	40/9/0 (82.6% F)	1 (2.0%)	6	42 (85.7%)
55–64	86	60.1 (2.6)	75/11/0 (87.2% F)	1 (1.16%)	6	82 (95.3%)
65–74	129	69.2 (2.7)	94/34/1 (72.9% F)	13 (10.1%)	9	124 (96.1%)
75–84	38	77.7 (2.8)	27/11/0 (71.1% F)	5 (13.2%)	5	38 (100%)
85–94	4	89.0 (0.8)	4/0/0 (100% F)	0 (0.0 %)	1	4 (100%)

*Note*. Further demographic information is presented in the Supplemental Material, including participants’ level of education (Table S1), all countries represented (Table S2) and data on the percentages of mono- versus bilingual participants (Table S9).

### Ethics

The study was approved by the Ethics Committee for Philosophy, Psychology and Language Sciences at the University of Edinburgh. After accessing relevant information about the study, all participants provided informed consent to participate before participating in the study.

### Transparency and openness

De-identified data and analytic code are available for viewing via the Open Science Framework (https://osf.io/kvja9/?view_only=f343fce9eccb44248fcaaa16e378b1c6). *R* Version 4.0.2 was used to analyze the data. The study design, hypothesis and analytic plan were not pre-registered. We report how we determined our sample size, any data exclusions, all manipulations and all measures in the methods section.

### Experimental tasks

#### Verbal memory single-task

Participants were asked to remember sequences of 6 or 7 letters from a set of 17 consonants, appearing sequentially at the screen centre. Each letter was shown for 750 ms, followed by a 250-ms blank screen. The sequence of letters was followed by a black circle that flashed on the screen for 10 s (750 ms on, 250 ms off). Participants were instructed to remember the letters in the order that they saw them, but keep their eyes on the circle and their fingers away from the keyboard for the duration of the 10-s period. Then, participants typed the letters using the keyboard in the order that they saw them. There were 10 trials, the first half of which required memory for 6 letters, and the other half for 7 letters.^3^ We used the average accuracy (i.e. the percentage of correctly recalled letters in the correct position) as the performance measure for our analyses. This task was adapted from Rhodes et al. (2019).

#### Visuo-spatial processing single-task

Participants were instructed that they would see two boxes with a gap between them and a bar below them. They had to decide as quickly and accurately as possible whether the bar would fit in the gap between the two boxes. They pressed ‘1’ on their keyboard for Yes (‘the bar does fit between the boxes’) and ‘0’ for No (‘the bar does not fit between the boxes’). In each trial, participants had 10 s to complete as many box-fit judgements as possible, and they completed a total of 10 trials. When they pressed the key, the bar moved towards the boxes and either went through the gap or flew off to the side, depending on whether it was a fit or no-fit trial. The black boxes flashed green when the participant got it right and red if they got it wrong. We used the average number of correct responses as the performance measure for our analyses. This task was adapted from Vergauwe et al. (2009).

#### Dual-task (Memory + Processing task)

This task was a combination of the aforementioned Memory and Processing tasks performed simultaneously. Participants completed the processing task in the 10-s interval between the presentation of the to-be-remembered letters but before the memory response. Thus, participants had to remember the letters while completing the processing task. Each participant completed 10 trials.

#### Working memory running span

Participants saw a sequence of digits appearing on the screen, presented one by one. Each sequence contained between 4 and 11 digits. When the sequence ended, participants had to report the four final digits (in the correct order) using the keyboard. The sequence length was random. For instance, they might get a sequence of 6 digits first, then 4, then 11. All participants saw one sequence of each length (between 4 and 11 digits), resulting in a total of 8 trials. There was no duplication of digits within a sequence, except at sequence length 11, in which one randomly selected digit was shown twice. Each digit was presented for 1,000 ms, followed by a 500 ms gap. We used the average accuracy (i.e. how many of the digits were recalled correctly) as the outcome measure for our analyses. This task was adapted from Pollack et al. (1959) and Bunting et al. (2006).

#### Silly sentences (working memory span)

Participants were asked to remember digits while also responding to true or false (‘Silly’) statements. First, a digit was presented for 1 s followed by a sentence that could be either True (e.g. ‘Bananas are Yellow’) or Silly (e.g. ‘Chairs are Liquid’). Participants responded as quickly and accurately as possible to whether the sentence was True or Silly (i.e. False) by clicking on either option on the screen. Then, a second digit appeared, which they also needed to remember, followed by a new sentence. At the end of a sequence, participants typed in all the memorized digits. This procedure was adaptive, such that all participants started at two digits (with two corresponding sentences). They performed two trials at this level. If they remembered all digits correctly, the task continued with an extra digit (and a corresponding sentence), up to a maximum level of seven digits (and corresponding sentences). If they did not correctly remember all digits at a given level, the task ended. We used the maximum level reached as the outcome measure for our analyses. This task was adapted from A. D. Baddeley et al. (1985) and Duff and Logie (2001).

### Deary-Liewald reaction time tasks

#### Simple reaction time task

In this task, one white square was positioned in the centre of a computer screen (Deary et al., 2011). A diagonal cross would appear within the square. Each time a cross appeared, participants responded by pressing the ‘1’ key as quickly as possible. Each cross remained on the screen until the key was pressed, after which it disappeared, and another cross appeared shortly after. The inter-stimulus interval (i.e. the time interval between each response and when the next cross appeared) ranged between 1 and 3 s and was randomized within these boundaries. Each participant completed 20 experimental trials. We used the average simple reaction times as the outcome measure for our analyses.

#### Choice reaction time task

Four white squares were positioned in a horizontal line across approximately the middle of the computer screen. Four keys on a standard computer keyboard corresponded to the different squares. The position of the keys corresponded to the position of the squares on the screen (the ‘*1*’ key corresponded to the square on the far left, the ‘*2*’ key to the square second from the left, the ‘9’ key to the square second from the right and the ‘0’ key to the square on the far right). In each trial, a diagonal cross appeared randomly in one of the squares, to which participants responded as quickly as possible by pressing the corresponding key on the keyboard. Each cross remained on the screen until one of the four keys was pressed, after which it disappeared, and another cross appeared shortly after. The inter-stimulus interval ranged between 1 and 3 s and was randomized within these boundaries. Each participant completed 40 experimental trials. We used the average reaction times for correct trials as the outcome measure for our analyses.

### Procedure

After consenting to participate and responding to a set of demographic questions, all participants started with either the Memory Single-Task or the Processing Single-Task (random order). After completing these two tasks, they performed the Dual-Task (Memory + Processing), followed by the Running Span, the Silly Sentences task and the Single and Choice RT tasks. At the end of the session, participants completed the Prospective and Retrospective Memory Questionnaire (Smith et al., 2000, data not reported here). After receiving instructions, participants started each task with a set of practice trials, for which they received performance feedback.

## Results

### Analytical approach

Throughout the study, we used a Bayesian approach to data analysis, which is argued by some to provide a stronger foundation for probabilistic inference than traditional null hypothesis significance testing (see Kruschke, 2011; Raftery, 1995; Wagenmakers, 2007). We apply a nomenclature in which *BF*_10_ refers to the Bayes Factor (*BF*) for the presence of an effect and *BF*_01_ refers to the absence of an effect, where *BF*_01_ = 1/*BF*_10_. When interpreting *BF*s, we rely on guidelines from van Doorn et al. (2021), in which a *BF* between 1 and 3 is considered *inconclusive* or *weak*, while a *BF* between 3 and 10 is considered *moderate*, and between 10 and 100 is considered *strong*. We refer to *BF*s >100 as *decisive* (Wetzels & Wagenmakers, 2012). However, these categorical verbal labels are subjective and should not be interpreted as definitive cut-off points (Tendeiro & Kiers, 2019; van Doorn et al., 2021).

### Does the magnitude of the dual-task cost change across the lifespan?

First, we explored dual-task costs across the age range. For these analyses, we used the *R* package *brms* (Bürkner, 2017, 2018). For memory, we explored the effect of age and task-type (Single vs. Dual) on memory performance (i.e. the average percentage of correctly recalled letters in the correct position). For processing, similar effects were explored on the number of correctly completed processing trials in the gap-fitting task. Participant identity was included as a random intercept to account for individual variation. We used the default priors and 10,000 iterations. We report the task performance parameter estimate (beta, *b*) and its 95% credible interval for age and task type. For each model, the credible interval (the values in square brackets) indicates the lower and upper bounds of the 95% credible interval of the posterior distribution for the parameter, indicating that given the data and our prior assumptions, there is a .95 probability that this interval encompasses the effect of beta. If this interval straddles 0, this suggests there was no credible effect of the specified factor on the outcome variable. In addition to examining the posterior distributions for the parameters, we also compared the model expected log predictive density using *k*-fold leave-one-out cross-validation (Vehtari et al., 2017), see Supplemental Material for details. Finally, we report the *BF* in favour of the model including the age by task-type interaction, to a model without this interaction, obtained using the *brms* ‘bayes_factor’ function, computing *BF*s from marginal likelihoods via bridge sampling (see Gronau et al., 2020).

#### Memory dual-task costs

**Figure 1.**
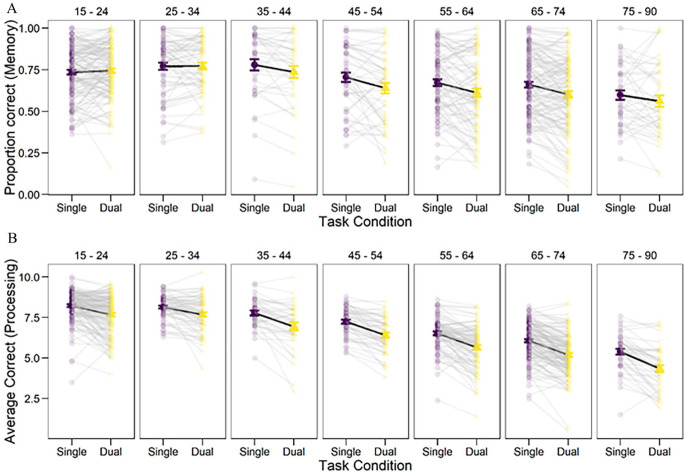
(A) Memory dual-task costs across the lifespan. (B) Processing dual-task costs across the lifespan. Error bars represent Standard Error. Grey lines and faded points represent individual data participants.

We compared five models (*M*_null_, *M*_age_, *M*_load_, *M*_age+load_, *M*_age×load_). The null model included only subject effects. We used a *k*-fold cross-validation model comparison and found that the best-fitting model was *M*_age×load_ (see Supplemental Material for details). The *BF* in favour of the interaction model (*M*_age×load_) was 4.7 × 10^3^ over a model not including this interaction (*M*_age+load_), suggesting that dual-task costs increased with age (see Figure 1A). Using this best-fitting model we found credible evidence for an age effect on memory (*b* = −.21; *SE* = 0.04, 95% CI [−0.29, −0.13]), and credible overall evidence that single memory performance was better than performance under processing load (*b* = 2.97; *SE* = 1.47, [0.11, 5.83]). Average single and dual task memory performance by age are summarized in Table 2.

**Table 2.** Average performance by age group.

Age group	Memory single (accuracy)	Memory dual (accuracy)	Processing single (correct responses)	Processing dual (correct responses)	Running span (accuracy)	Silly sentences (maximum level reached)	Simple RT (in ms)	Choice RT (for correct trials; in ms)
15–24	0.74 (0.16)	0.74 (0.16)	8.2 (0.9)	7.7 (1.2)	0.51 (0.09)	4.3 (1.7)	291 (50)	423 (72)
25–34	0.77 (0.18)	0.77 (0.16)	8.1 (0.7)	7.7 (1.1)	0.52 (0.08)	5.0 (1.8)	303 (46)	463 (82)
35–44	0.78 (0.21)	0.74 (0.22)	7.8 (0.9)	6.9 (1.5)	0.48 (0.11)	4.8 (1.7)	310 (46)	509 (100)
45–54	0.70 (0.20)	0.64 (0.22)	7.2 (0.9)	6.4 (1.1)	0.48 (0.11)	4.9 (1.8)	299 (34)	533 (120)
55–64	0.67 (0.19)	0.61 (0.23)	6.5 (1.0)	5.7 (1.3)	0.48 (0.11)	4.8 (1.5)	314 (43)	616 (156)
65–74	0.66 (0.19)	0.60 (0.22)	6.1 (1.2)	5.2 (1.3)	0.46 (0.10)	4.7 (1.7)	324 (50)	664 (168)
75–90	0.60 (0.18)	0.56 (0.22)	5.4 (1.2)	4.3 (1.4)	0.45 (0.10)	3.9 (1.6)	355 (90)	711 (147)

*Note*. Mean values by age group, values in parenthesis represent the standard deviation.

However, the age × task-type (single or dual) interaction could be driven by younger adults performing at near-ceiling levels in the Single-Task condition, to a greater degree than older adults. To test this possibility, we excluded a total of *N* = 90 participants who performed better than 90% correct in the single memory task (exclusions by age group: 15–24, *N* = 20; 25–34, *N* = 16; 35–44, *N* = 14; 45–54, *N* = 8; 55–64, *N* = 13; 65–74, *N* = 18; 75–84, *N* = 1). The pattern of results was the same in this data set (the age × task-type model was favoured over the model without the interaction term by *BF* = 3.7 × 10^3^), suggesting that ceiling effects did not drive this effect (see Supplemental Material for details). Finally, similar results were found using performance over 85% as a more stringent cut-off point for ceiling effects (the age × task-type model was favoured over the model without the interaction term by *BF* = 2.1 × 10^3^, *N* = 386).

#### Processing dual-task costs

We conducted a similar analysis for the processing data. Similar to the memory analysis reported above, the best-fitting model included the age × task-type interaction (*M*_age×load_, see Supplemental Material for details). The *BF* in favour of the model including the age × task-type interaction (*M*_age×load_) factor was 18.4, over a model not including this factor (*M*_age+load_), suggesting that the dual-task costs were greater in older adults (see Figure 1B). This best-fitting interaction model provided credible evidence that single processing performance was better than performance under memory load (*b* = −.38; *SE* = 0.10, 95% CI [−0.57, −0.18]), and that performance declined with age (*b* = −.05; *SE* = 0.00, [−0.05, −0.04]). Overall, these results suggest that processing dual-task costs was greater in older than in younger participants (See Figure 1B). Average single and dual-task processing performance by age is summarized in Table 2.

Next, to rule out potential confounds caused by ceiling effects, we excluded 84 participants who performed at the top range in the single processing task, defined as correctly completing an average of 8.5 processing sequences or more (exclusions by age group: 15–24, *N* = 52; 25–34, *N* = 21; 35–44, *N* = 8; 45–54, *N* = 2; 55–64, *N* = 1). Evidence for a task-by-age interaction was also found in this data set (*BF* = 74.1, in favour of the age × task-type model), suggesting that Single-Task ceiling effects did not drive this effect (see Supplemental Material for details). A similar analysis, which excluded participants with an average score of more than 8 correct responses in the single processing task as an even more conservative cut-off point against potential ceiling effects, found inconclusive evidence for the age × task-type model (*BF* = 1.3, *N* = 363). However, we note that this task, theoretically, did not have a performance ceiling. The highest number of correct responses in an individual processing episode was 12, and the highest attempted number of responses (regardless of accuracy) was 16.

Finally, we note that average processing response times between the Single and Dual tasks appeared especially large for the very first processing judgement (Single = 949.5 ms, Dual = 1744.8 ms), compared to the subsequent processing episodes (e.g. average RTs for the second processing judgements were: Single = 752.3 ms, Dual = 892.9 ms, see Table S7), which suggests that the processing dual task cost may be at least partly driven by slowed initiation of the processing run, as participants are disengaging from the memory task.

#### General dual-task ability

**Figure 2.**
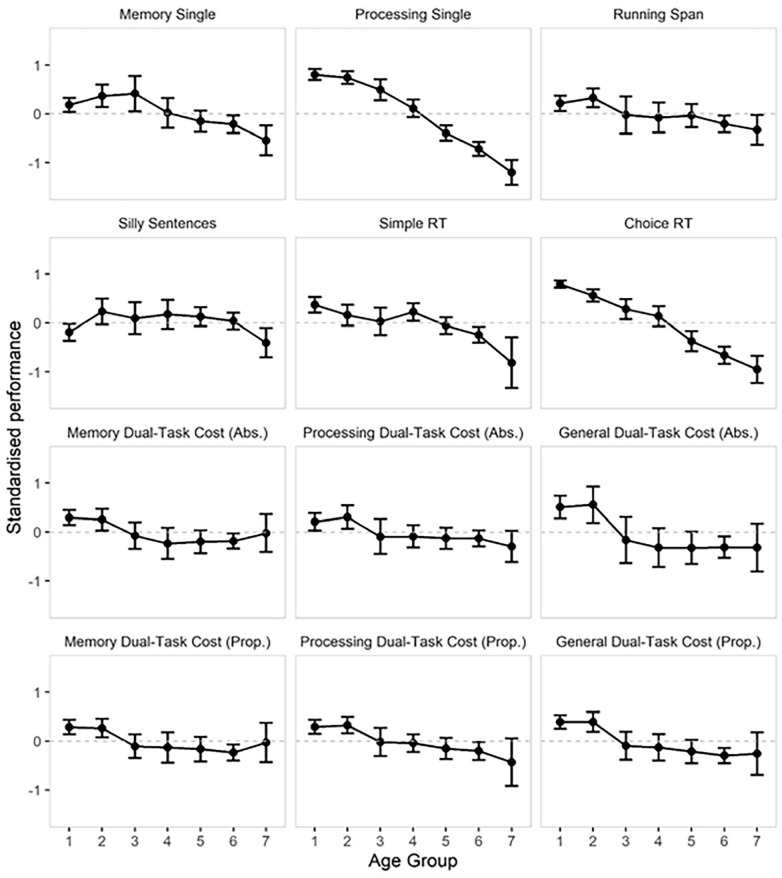
Average standardized (*z*-scores) performance by age group and task. Error bars represent 95% Confidence Intervals. Dual-Task Cost panels represent the standardized relative dual-task costs (i.e. performance decline between single and dual-task versions of each task), reverse scored (multiplied by −1) so that higher values represent a lower cost. Abs. = Absolute cost (Single Task Performance − Dual Task Performance) and Prop. = Proportional cost (Dual Task Performance/Single Task Performance). Scores for both RT measures (Simple and Choice) were also reversed, so higher values represent faster RTs. The ages in different age groups were as follows: 1 = 15 to 24 years, 2 = 25 to 34 years, 3 = 35 to 44 years, 4 = 45 to 54 years, 5 = 55 to 64 years, 6 = 65 to 74 years and 7 = 75 to 90 years.

Next, we used a combined Dual-Task Ability measure (i.e. the sum of each participant’s standardized memory and processing dual-task costs). This allowed us to explore dual-task costs while accounting for potential trade-offs (e.g. participants may choose to focus less on one of the tasks in the dual-task condition, see Belletier et al., 2023). Standardization is necessary because the measures are on different scales. We found evidence for a positive correlation between age and dual-task cost (Bayesian correlation, ρ = .25, *BF*_10_ = 5.7 × 10^6^). Excluding all the participants with single memory accuracy >90% and/or a processing score of 8.5 left a sample of *N* = 386 and did not change the pattern of this result (Bayesian correlation, ρ = .27, *BF*_10_ = 3.0 × 10^5^, See Figure 2).

### Do memory and processing abilities decline at the same rate with age?

Next, we explored whether performance on memory and processing tasks declined at similar rates across the lifespan. The average performance by age group is presented in Table 2. We excluded dual-task performance and dual-task costs for these analyses. We included the following six measures: Memory Single-task, Processing Single-task, Running Span, Silly Sentences, Simple RT and Choice RT. The two RT measures were reverse coded (multiplied by −1) so that higher *z*-scores reflect better performance, making them comparable to the other tasks. We included task type (categorical) and age (continuous) as factors, and performance (*z*-scores) as the outcome variable in a Bayesian regression model, and participant ID as a random slope. The *BF* in favour of the interaction model (*M*_age×task_) was 8.66 × 10^52^, over a model not including the interaction (*M*_age+task_). Performance on the Processing Single-Task and the Choice RT tasks appeared to suffer the most pronounced age-related decline (see Table 2 and Figure 2). We compared only Simple RT and Choice RT in a separate follow-up analysis and found decisive evidence for a model including the task × age interaction (*BF* = 6.8 × 10^10^), compared to a model including the main effects of task and age, but no interaction, suggesting a steeper age-related decline in the Choice, compared to the Simple RT task (see Table 2 and Figure 2).

### Is cognitive decline continuous across the adult lifespan?

To explore the continuity of performance levels, we used a piecewise approach, in which we compared the average performance in one age group (e.g. 15–24 years), to the performance in *all* older participants than those in this age group (e.g. aged 25 or more). The results of these analyses are presented in Table 3, and performance decline rates are presented in Figure 2. For some of the tasks, an incremental lifespan change in performance can be observed, such that the average performance differed between each group contrast (Processing Single, Complex RT and Simple RT; although with some inconclusive *BF*s). For the Silly Sentences task (working memory span), evidence for age differences was generally not found between any of the age groups. For Single Memory performance, evidence for a continued decline with age was mixed or inconclusive after the age of 44. For dual-task costs and Running Span performance, the analyses suggested that after participants’ mid-30s (25–34 years), there were generally no further performance declines. In these measures, the youngest-young groups (aged 15–34 years) stood out as performing better than all older participants, without further decline after this age cut-off. This suggests that the overall age difference in dual-task costs described above was driven by changes between the youngest-young adults and all participants older than 35 years, rather than by continuous decline with increasing age.

**Table 3.** Bayes Factor evidence participants in a given age group performed differently from *all older participants* in the study (i.e. all participants older than the participants in the specified age group).

Age group	Memory single	Processing single	Running span	Silly sentences	Simple RT	Complex RT	Memory dual-task (relative/proportional)	Processing dual-task (relative/proportional)	General dual-task (relative/proportional)
15–24	*1.84*	**7.3 × 10** ^25^	**4.9**	*2.5*	**1.5 × 10** ^4^	**1.2 × 10** ^25^	**195.4/134.8**	**5.1/176.4**	**4.1 × 10** ^3^ **/6.3 × 10** ^4^
25–34	**62.4**	**8.8 × 10** ^18^	**36.0**	*0.42*	*2.4*	**3.5 × 10** ^10^	**10.7/13.3**	**27.7/73.6**	**1.4 × 10** ^3^ **/2.0 × 10** ^3^
35–44	**39.0**	**1.7 × 10** ^9^	0.24	0.20	*0.39*	**2.6 × 10** ^3^	0.21/0.20	0.19/0.28	0.22/0.25
45–54	*0.61*	**1.2 × 10** ^7^	0.19	0.32	**16.4**	**1.3 × 10** ^4^	0.18/0.17	0.18/0.29	0.16/0.22
55–64	0.24	**372.9**	*0.41*	*0.45*	*1.9*	**5.0**	0.15/0.15	0.15/0.18	0.14/0.16
65–74	*0.87*	**24.5**	0.23	**3.5**	**5.9**	*0.63*	0.27/0.33	0.29/0.31	0.18/0.19

*Note*. Values in bold represent evidence in favour of an age difference, italics represent inconclusive evidence, and underscored represents evidence against an age difference. The *Dual-Task* measures represent the standardized relative dual-task costs (i.e. how much performance declined between the single and dual-task versions of a given task; values to the left, and proportional differences between the single and dual-task versions; values to the right).

When assessing dual-task costs, it is important to consider whether the units of measurement that we are using are equivalent across performance levels and age groups (for a discussion, see Loftus, 1978; Wagenmakers et al., 2012). For example, a dual-task memory drop of one letter is *proportionally* more noteworthy for a participant whose overall single-task memory span is three letters than for a participant with a single span of six letters. However, this relies on the assumption that the underlying scale for high and low scoring participants is linear and equivalent (for a discussion, see Perfect & Maylor, 2000). It is also important to ensure that measures are equivalent psychologically. Older participants might perform a given task in a different way from younger participants, so the task could be measuring a different cognitive ability in different age groups, and comparing proportional scores could be misleading. For example, Forsberg et al. (2020) showed that older people might use verbal strategies to do a visual memory task (for discussions see Logie, 2018, 2023; P. M. A. Rabbitt, 2000). To address these contrasting views, we used two alternative measures of dual-task ability. First, *Standardized Absolute Dual Task Cost* (i.e. Single Task Performance − Dual Task Performance), which captures the raw performance difference (e.g. how many more gap-task processing episodes a participant successfully completed in the single, compared to the dual task condition). Second, *Proportional Dual Task costs* quantify the *proportion* of performance that is sacrificed under a dual-task demand (Dual Task Performance/Single Task Performance). Moreover, we also looked at dual-task costs separately for memory and processing outcome measures, and as a *combined*, general measure, to help account for potential strategic shifts (e.g. under dual-task load, people may decide to choose to focus on one task at the detriment of the other, and there may be age differences in such preferences). Notably, across all six measures of dual-task ability, differences across the adult lifespan were characterized by better dual task ability in the two youngest groups (14–34 years), but from that point onwards, dual-task ability appeared consistent – that is, both absolute and proportional dual-task costs were equivalent in the middle-aged and older adult participant groups (see Figure 2).

To rule out dual-task cost confounds caused by potential ceiling performance, we excluded all participants who were close to ceiling-level performance in either the memory or the processing single task (or both), as specified above, leaving a sample of 386 participants. Similar patterns emerged, such that the 15 to 24 and 25 to 34 age groups both differed from all participants older than them (*BF*_10_ = 408.6 and *BF*_10_ = 1.5 × 10^3^ respectively), while evidence *against* an age effect was found when comparing the 35 to 44 age group (and all subsequent groups) to all participants who were older than them (all *BF*_10_ ⩽ 0.30). Finally, we note that the proportion of monolingual English speakers was higher in the older age groups (see Table 1 and Table S9). To address this potential confound, we analyzed the data, including only monolingual, native English speakers (*N* = 367), and found similar patterns of results (see Tables S10 and S11).

### Correlations

Task correlations (in the complete participant sample and in younger and older participants, respectively) are reported in Tables S4 and S6. We observed significant (*p* < .001) correlations between different memory tasks (including between Memory Single, Memory Dual, Running Span and Silly Sentences), as well as between different processing tasks (Processing Single, Processing Dual, Simple and Choice RT). There were also some (generally weaker) correlations between different types of tasks (see Tables S4 and S6). Finally, in Table S5, we report correlations between odd and even number trials (‘split-half reliability’) in all participants (*N* = 539) for all tasks except the Silly Sentences task (for which such an analysis was not appropriate, as the adaptive nature of this task meant that task difficulty and continuation depended on performance in prior trials). These correlations were all statistically significant (*p* < .05) and ranged from .11 (Simple RT) to .71 (Processing Single), with reliabilities between .20 and .83.

## Discussion

We explored how memory, processing, and dual-task ability vary across the lifespan, using a set of 7 tasks thought to tap these abilities, completed by 539 participants aged between 15 and 90 years old. Our key results were as follows. First, we found evidence that overall age-related changes in dual-task costs (i.e. verbal memory performance when participants had to complete a concurrent visuo-spatial processing task and vice versa) were not continuous and incremental. Dual task costs were larger in participants aged 35 to 90 years than in participants aged 15 to 34 years. Stepwise analyses suggested that there was no detectable change in dual task costs between participants in early mid-life and those more advanced in age. Additional analyses confirmed that these patterns were not driven by single-task ceiling performance in younger adults. Processing abilities, particularly on tasks requiring a choice combined with a speeded response, appeared to decline at a steeper rate with age than performance on tasks requiring recall or simple processing. Finally, different memory abilities appeared to change at different rates across age. A measure of working memory span (the Silly Sentences Task) showed no evidence of age-related decline, whereas verbal memory span for letters and running memory span showed decline between early adulthood and mid-life, but inconclusive or no further decline after mid-life through to old age. We discuss the theoretical implications of these findings below.

### Lifespan dual-task costs: trajectories and mechanisms

Our results indicate that both processing and memory performance were poorer under dual-task compared with single-task conditions, and crucially, that this impairment was greater for participants older than 35 years, compared with younger participants. Follow-up analyses suggested that this result was not driven by ceiling effects in either of the single tasks. Also, we found evidence for age-related dual-task costs using a combined measure of standardized dual-task costs, suggesting that this result was not modulated by age differences in the tendency to focus on one task at the expense of the other (see Lindenberger et al., 2000; Navon & Gopher, 1979; Rhodes et al., 2019). The observed increased dual-task cost with age is consistent with other findings using similar paradigms (Bier et al., 2017; Jaroslawska et al., 2021, Rhodes et al., 2019, 2021), and, more broadly, with those studies reporting evidence that older adults are comparatively more impaired than young adults when required to coordinate concurrent cognitive activities (Craik, 1977; Mayr & Kliegl, 1993; Salthouse, 1990). However, by exploring age as a continuous variable rather than a binary grouping variable as in many previous studies, we observed larger relative dual-task costs in participants in their mid-thirties, compared to the dual-task costs of their younger counterparts. The average standardized dual-task costs appeared remarkably consistent after the age of 35 (see Figure 2). These findings appear partially aligned with recent evidence of nonlinear patterns in molecular markers of ageing, including a recent study that has observed two periods of substantial molecular dysregulation occurring at the ages of 44 and 60 years (Shen et al., 2024). These time points may also correspond to changes in common lifestyle factors, such as professional activity or caring responsibilities. Moreover, this seemingly non-linear trajectory highlights the benefit of studying the complete adult lifespan instead of only comparing younger and older adults. Our findings suggest that instead of discussing ‘dual-task deficits in older adults’, reframing the discussion to ‘dual-task benefits in very-young adults’ appears more accurate. This finding offers a possible hypothesis for testing in future research that could resolve some of the previous contrasting results of either the presence or the absence of age-related dual-task costs. Whether or not an age-related dual task cost is observed may depend, at least in part, on the age ranges of the groups being compared. There may also be substantial variability among the performance levels of either group that could undermine age group differences. This is less of a problem when age is a continuous variable rather than a binary grouping variable, and with a reasonably large sample size.

One limitation of our study is that, because of constraints on collecting data online from most of our participants, single task demand levels for memory and processing were not adjusted (titrated) for the span of each participant to equate single task performance across participants of different ages. As noted in the Jaroslawska and Rhodes (2019) meta-analysis, in most studies, when such titration of single task demand is used, the age-related decline in dual task cost is very small or zero. Rhodes et al. (2019, 2021) reported age-related dual task costs even when task demands were titrated, although both tasks involved verbal processes (verbal memory and arithmetic verification), and there was no age-related decline in prioritizing one or other task. Future studies might explore when titration does or does not modulate dual task performance across participants. However, our data show clearly that when single task demand is not titrated, dual-task performance does not appear to decline beyond early middle age, and that performance on different cognitive tasks changes at different rates across age.

#### Mechanisms of dual-task costs and benefits

Our finding of a youngest-young adult dual-task benefit may provide interesting insights into the potential causes of these dual-task costs – especially when comparing this performance trajectory to that of the other cognitive tasks included in this study. Various explanations have been proposed to explain why older adults might have a deficit in coordinating competing demands in dual-task paradigms (see a recent discussion by Naveh-Benjamin & Cowan, 2023). Age-related dual-task effects could be explained by older adults’ slowed speed of processing which is a crucial part of working memory performance in young adults (see Barrouillet et al., 2004, 2007; Barrouillet & Camos, 2014), due to the well-known effects of ageing on processing speed with slowing observed as early as the mid-20s (Cerella, 1985; P. Rabbitt & Goward, 1994; Salthouse, 1996). If adults in their 30s and older take longer to complete each processing event, that would leave less time to refresh items in memory (Camos et al., 2009), thus increasing the detrimental impact of the dual-task condition. However, the age-related dual-task cost has been shown to be present even when older adults (aged 67–80 years) are given more time for memory encoding or for processing (Rhodes et al., 2021). In the current study, performance on the other tasks requiring rapid processing (Single Processing, and Simple and Choice RT) showed a relatively steady, continuous decline in performance across the lifespan, not only in those over 67 years. However, given the Rhodes et al. (2021) results, this age-related slowing is unlikely to have modulated age differences in relative dual-task costs. Moreover, we would have expected the dual-task cost patterns across age to follow the continuous pattern of decline observed for the processing and RT tasks, which was not the case.

Further, older adults may suffer a more pronounced dual-task cost due to their reduced peripheral non-attentional processing resources (Greene et al., 2020). Performance on the Single Memory task may reflect the combination of a limited capacity general-purpose central resource *and* limited capacity, domain-specific peripheral resources (see Camos & Barrouillet, 2011; Cowan et al., 2014; Logie, 2011, 2023). For younger adults, some researchers have argued that small or absent dual task impairments, relative to single task performance, have been found because each task uses a different domain-specific, limited capacity peripheral resource that can operate in parallel, but with a small general cognitive load from the requirement to perform two tasks concurrently (e.g. A. Baddeley et al., 1986; Cocchini et al., 2002; Logie, 1995, 2011, 2023; Logie et al., 2004). Alternatively, one task might rely more on a general-purpose central resource, while the other task relies on a peripheral resource (e.g. Baddeley & Hitch, 1974; Barrouillet et al., 2021; Camos et al., 2009; Cowan et al., 2014). Our current data cannot distinguish between these accounts, but whichever of these accounts might apply to the performance of young adults, suppose that from early middle age onwards, adults use a greater proportion of their limited capacity general purpose central resources, and have depleted capacity for their domain-specific peripheral resources to maintain their single-task performance. In that case, they might have little to spare to coordinate the dual-task, which might require switching between tasks, perhaps exacerbating their dual-task cost. This could explain why age-related dual-task deficits are often not observed when task difficulty is adjusted based on single-task performance (Jaroslawska & Rhodes, 2019). In our study, the Running Span task appeared to follow the dual-task cost lifespan trajectory most closely. Perhaps the use of central resources for attentional focusing on relevant items (Cowan, 1988, 2010; Morey & Bieler, 2013) which is required to manage the continuous updating demands in this task, or the need to focus attention promptly on the stimulus stream when it ends, begins to decline in early adulthood, but then stays relatively intact through middle and older age. Similar central resource attentional mechanisms tapped in this task, with less capacity in peripheral resources, may contribute to participants’ relative dual-task costs. This offers a possible set of hypotheses to explore in future studies.

Another suggestion that might explain age differences in relative dual-task costs is that by early middle age, adults may have adopted a range of different strategies (see discussions in Logie, 2018, 2023) from those in early adulthood for the Single-Task condition, and these strategies might be disrupted in the dual-task condition. For example, older adults seem to favour verbal rehearsal of memoranda when possible, even for tasks assumed to measure visual memory (Forsberg et al., 2019, 2020). While such a strategy might help older adults boost their Single-Memory task performance, it might not be effective in the dual-task condition (see Belletier et al., 2023). In the current study, participants completed both single tasks prior to the dual task, which might have resulted in a practice effect. However, this is only likely to have benefitted older adults if they had more practice than younger participants. Participants of all ages had the same amount of practice, so this is an unlikely explanation for the lack of age-related dual task decline beyond early middle age (although see Burger et al, 2020; Luszcz & Hinton, 1993).

Observations that age-related dual-task deficits depend heavily on paradigm differences (De Ribaupierre & Ludwig, 2003; Riby et al., 2004) support the general idea that older adults may use more – or different – resources or techniques for certain single-tasks, which are disrupted by certain types of dual-task demands. Looking at the differential trajectories of decline, it seems more clearly linear for tasks in which verbal rehearsal is unlikely (Single Processing and the two RT tasks). Literature on strategic shifts across the lifespan (particularly, in middle age) appears sparse, but is needed to help determine whether the trajectory differences observed here may be driven by age differences in strategic preferences rather than shifts in cognitive ability per se (Logie, 2011, 2018, 2023).

### Memory and processing abilities decline at different rates with age

We found that performance in complex processing tasks seemed to change more across age than performance in memory or simpler processing tasks. Specifically, Processing Single-Task and Choice RT saw the steepest age-related decline (see Figure 2). These two tasks both required a decision (does it fit in the gap, or which key to press), combined with a speeded response. The idea of age-related decline in response speed – or processing speed more broadly – is well established (e.g. Der & Deary, 2006; Nettelbeck & Rabbitt, 1992, but see von Krause et al. 2022). Choice RT performance declined more rapidly than Single RT performance, suggesting that the processing choice was specifically affected by age-related decline in general response speed. These findings could have consequences for the dual-task literature, as we might expect larger age-related dual-task costs if the processing task requires a choice rather than a simple response. In contrast, performance on the measure of working memory span (Silly Sentences task) appeared relatively intact across the lifespan. This task included both memory (digit recall) and a concurrent processing task (responding to semantic statements) and could be considered a dual-task paradigm. This task was discontinued as soon as participants forgot one digit, which made it especially sensitive to potential inattentiveness. This task was also self-paced and required semantic knowledge, which might have benefitted from greater lifetime accumulated semantic knowledge in the older adults (e.g. Jaroslawska et al, 2021; Mohanty et al., 2016). Moreover, we note that measurement using complex span tasks usually involves a preliminary phase where time to complete processing is measured and then applied on an individual basis (e.g. Picture span, Hicks et al., 2016). This was not applied in our procedure, and it is therefore possible that participants used the processing episodes as an opportunity to rehearse memoranda by momentarily stalling before proceeding in the trial, which may also explain the lack of age differences observed in this measure.

#### Potential cohort and participant group effects

Finally, there are potential limitations from using cross-sectional data to explore trajectories of cognitive decline, including potential cohort effects (Salthouse, 2009). For example, access to digital technologies in childhood and adolescence may play a role in how participants approach cognitive tasks. Yet, some suggest that the relationship between video game experience and fundamental cognitive abilities is weak or nonexistent (e.g. Unsworth et al., 2015; von Bastian et al., 2022, but see also Waris et al., 2019). However, while cohort differences might account for contrasting results between very young adults (e.g. 15–34 year olds) and 65 to 90 year olds, this is an unlikely explanation for the differential pattern of decline across tasks, particularly in the middle age range where the age differences are not large. As in previous large-sample lifespan cohort studies (e.g. Johnson et al., 2010), participants aged 25 to 34 years and those aged 35 to 44 are likely to have had similar educational and life experiences, yet show differences in performance on some tasks. Future research using large, well balanced samples, and with age as a continuous, not binary, variable, could have potential to explore whether the mid-life stability observed in some cognitive abilities in our study (dual-task and updating ability) but not others (e.g. processing) are driven by environmental cohort differences that might result in use of alternative strategies by different participants, or by more fundamental age-related changes in some, but not all cognitive processes. A longitudinal approach in future research would have limited potential to help better understand the cognitive trajectories of dual-task performance. As mentioned in the introduction, there are caveats for longitudinal studies of possible practice effects, drop-out rates (e.g. P. Rabbitt et al., 2004), and tasks possibly becoming outdated with respect to theory development over the multiple decades for the study.

Given that our data collection method involved advertising the study to anyone who was interested in participating, we were not able to control the number of participants in each age group, or match participants in different age groups based on their demographics. Thus, larger numbers of participants are seen in the 15 to 24 and the 55 to 64 age groups, relative to the other age groups (see Table 1). The homogeneity of country of residence varied between age groups, and the proportion of monolingual English speakers was higher in the older age groups (see Table 1 and Table S9). As such, there may be differences in how representative participants in the different age groups are of the general population. However, given the different trajectories between different tasks shown in previous studies with different and larger samples (e.g. Johnson et al., 2010; Maylor & Logie, 2010; Park et al., 2002) as well as in the current study (e.g. continuous, linear decline for Simple RT, and no age differences for the Silly Sentences task), it seems unlikely that the observed age trajectories in cognitive performance can be fully explained by factors like differences in motivation to follow task instructions, socioeconomic status, access to education, or the impact of the COVID-19 pandemic on participants in different groups. Control analyses including only monolingual, native English-speakers generally found similar patterns. Still, the varying density of participants in the different age groups presents a limitation, for the current study both due to potential confounds from group effects, and also due to relatively sparser data in some age groups. Finally, we note that our sample was predominately (77.8%) female. Females were overrepresented to similar extents across the age groups (see Table 1), although some prior research has reported faster memory decline in men than in women (e.g. Bloomberg et al., 2021). Results, especially those related to the more novel finding of a non-linear trajectory for dual-task costs, should be replicated in future studies with a more evenly distributed sample, to confirm their generalisability across genders.

### Conclusion

Our results suggest that abilities that support memory, processing and dual-task performance, decline at different rates as we age. These results reject the ‘Dull Hypothesis’ of ageing (Perfect & Maylor, 2000), the notion that human ageing causes uniform decline in all mental activities, given the observed differential decline of memory, processing and dual-task ability. The results suggest that age-related dual-task decline occurs by around age 35 but stays remarkably intact throughout middle and older age. Some of the findings may inform attempts to design environments that are easier to navigate as we age. For example, tasks that may be especially taxing for older adults involve combining a decision with a fast response. Whereas, while compared to very young adults, people over the age of 35 years may perform more poorly when asked to dual-task or multi-task (i.e. keep information in mind while also performing some type of processing task), our results indicate that this ability may stay relatively stable throughout middle and older age.

## Supplemental Material

sj-docx-1-qjp-10.1177_17470218251351307 – Supplemental material for Different measures of working memory decline at different rates across adult ageing and dual task costs plateau in mid-life Supplemental material, sj-docx-1-qjp-10.1177_17470218251351307 for Different measures of working memory decline at different rates across adult ageing and dual task costs plateau in mid-life by Alicia Forsberg, Clément Belletier, Agnieszka Graham, Stephen Rhodes, Pierre Barrouillet, Valérie Camos, Nelson Cowan, Moshe Naveh-Benjamin and Robert H Logie in Quarterly Journal of Experimental Psychology

## Notes

1. The full data set from 408,938 participants across a range of working memory and lifestyle measures is available on the Open Science Framework (Logie et al., 2020).

2. A Bayesian analysis using standardized scores for each of the experimental tasks found evidence against a performance difference between in-lab and online participation (*BF*_01_ = 3.2).

3. Thirty-nine participants completed all 10 trials with 6 letters, in both the single- and dual-task conditions. After these participants, we changed the programme so that half of the trials included 7 letters for all subsequent 500 participants, as a precaution against ceiling effects.
