# Supplementary material for: Different measures of working memory decline at different rates across adult ageing and dual task costs plateau in mid-life
Source: Q J Exp Psychol (Hove). 2025 Jun 6;79(2):462–79. doi: 10.1177/17470218251351307 (PMC12796017; doi:10.1177/17470218251351307)
Supplement: sj-docx-1-qjp-10.1177_17470218251351307 – Supplemental material for Different measures of working memory decline at different rates across adult ageing and dual task costs plateau in mid-life [file sj-docx-1-qjp-10.1177_17470218251351307.docx]

Supplementary Material for:

**Different measures of working memory decline at different rates across adult ageing, and dual task costs plateau in mid life**

Alicia Forsberg^1^, Clément Belletier^2^, Agnieszka Graham^3^, Stephen Rhodes^4^, Pierre Barrouillet^5^, Valérie Camos^6^, Nelson Cowan^7^, Moshe Naveh-Benjamin^7^, Robert H Logie^8^

1 The University of Sheffield, UK

2 Université Clermont Auvergne, France

3 Queen's University Belfast, UK

4 Urology Institute, University Hospitals Cleveland Medical Center, USA

5 University of Geneva, Switzerland

6 University of Fribourg, Switzerland

7 Department of Psychological Sciences, University of Missouri, US

8 The University of Edinburgh, UK

**Author note:** Correspondence regarding this article should be addressed to Alicia Forsberg, University of Sheffield, UK, email: [a.forsberg@sheffield.ac.uk](mailto:a.forsberg@sheffield.ac.uk) or Robert H Logie, University of Edinburgh, UK, email: [rlogie@ed.ac.uk](mailto:rlogie@ed.ac.uk).

1. **Additional Demographics**

We present the self-reported levels of education by age group in Table S1, and a list on the countries of residence presented in Table S2.

1. **Model comparisons: Dual-task costs**

We compared *brms* model expected log predictive density (ELPD) using k-fold leave-one-one cross-validation (Vehtari et al., 2017). In this procedure, each model was refitted 10 times, each time leaving out one 10^th^ of the original data. Using this form of cross-validation, we obtain Bayesian LOO ELPD for each model; the theoretical expected log pointwise predictive density for a new dataset. We report the differences in the expected predictive accuracy of different models by the difference in the ELPD (see Table S3). The most accurate model is presented in the first row, with and ELPD of 0 (i.e., it does not differ from itself), and subsequent ELPD difference values represents the difference between this preferred model and each subsequent model.

*Table S1*. Levels of education by age group

| **Age Group** | **Education** | ***N*** |
| --- | --- | --- |
| 15 - 24 | High School | 64 |
|  | Primary School | 1 |
|  | University first degree | 43 |
|  | University Masters or PhD | 20 |
|  | Vocational College | 4 |
| 25 - 34 | Primary School | 1 |
|  | University first degree | 20 |
|  | University Masters or PhD | 39 |
|  | Vocational College | 4 |
| 35 - 44 | High School | 1 |
|  | University first degree | 8 |
|  | University Masters or PhD | 21 |
|  | Vocational College | 7 |
| 45 - 54 | High School | 11 |
|  | University first degree | 18 |
|  | University Masters or PhD | 9 |
|  | Vocational College | 11 |
| 55 - 64 | High School | 11 |
|  | University first degree | 34 |
|  | University Masters or PhD | 9 |
|  | Vocational College | 32 |
| 65 - 74 | High School | 29 |
|  | University first degree | 47 |
|  | University Masters or PhD | 22 |
|  | Vocational College | 31 |
| 75 - 84 | High School | 12 |
|  | University first degree | 11 |
|  | University Masters or PhD | 5 |
|  | Vocational College | 10 |
| 85 - 94 | High School | 2 |
|  | University first degree | 2 |

*Table S2*. Countries represented

| **Country** | ***N*** |
| --- | --- |
| Australia | 16 |
| Barbados | 1 |
| Bulgaria | 1 |
| Canada | 57 |
| Chile | 1 |
| China | 13 |
| Croatia | 3 |
| Finland | 1 |
| France | 2 |
| Germany | 4 |
| Hong Kong | 1 |
| Hungary | 2 |
| India | 4 |
| Iran, Islamic Republic of | 1 |
| Ireland | 1 |
| Italy | 2 |
| Japan | 1 |
| Jordan | 1 |
| Macedonia, The Former Yugoslav Republic of | 1 |
| Malaysia | 3 |
| Mexico | 1 |
| Netherlands | 1 |
| New Zealand | 16 |
| Nigeria | 1 |
| None | 2 |
| Poland | 1 |
| Romania | 1 |
| Russian Federation | 1 |
| Saudi Arabia | 1 |
| Senegal | 1 |
| Singapore | 2 |
| Spain | 2 |
| Sweden | 16 |
| Turkey | 2 |
| United Kingdom | 356 |
| United States | 19 |

*Table S3*. Dual-Task model comparisons

|  | **ELPD difference** | **SE difference** |
| --- | --- | --- |
| **Memory** |  |  |
| M_age×load_ | 0.0 | 0.0 |
| M_age+load_ | -9.5 | 14.4 |
| M_load_ | -10.1 | 15.3 |
| M_age_ | -15.8 | 15.9 |
| M_null_ | -29.6 | 16.4 |
| **Processing** |  |  |
| M_age×load_ | 0.0 | 0.0 |
| M_age+load_ | -11.0 | 11.0 |
| M_load_ | -107.6 | 19.7 |
| M_age_ | -217.5 | 19.6 |
| M_null_ | -310.8 | 22.5 |
| **Memory (Single Ceiling Removed)** |  |  |
| M_age×load_ | 0.0 | 0.0 |
| M_age+load_ | -26.8 | 10.8 |
| M_age_ | -26.9 | 10.5 |
| M_load_ | -27.5 | 10.8 |
| M_null_ | -44.8 | 13.5 |
| **Processing (Single Ceiling Removed)** |  |  |
| M_age×load_ | 0.0 | 0.0 |
| M_age+load_ | -13.8 | 12.7 |
| M_load_ | -70.6 | 16.3 |
| M_age_ | -173.7 | 17.8 |
| M_null_ | -242.4 | 20.6 |

*Note*. The best fitting model is presented first, all subsequent ELPD different scores is between the best-fitting model and the comparison model. Values obtained using ‘loo_compare,’ criterion = "kfold".

*Table S4.* Correlations (A). in younger vs. older adults (B) in all participants.

A. Correlations in younger (upper right, *N* = 233) and older (lower left, bold, *N* = 306)

|  | **Memory Single** | | | **Memory Dual** | | **Processing Single** | **Processing Dual** | **Running Span** | **Silly Sentences** | **Simple RT** | **Choice RT** | **Dual-task cost** |
| --- | --- | --- | --- | --- | --- | --- | --- | --- | --- | --- | --- | --- |
| **Memory Single** | **1** | | | 0.74* | | 0.15 | 0.16 | 0.51* | 0.34* | -0.08 | -0.09 | 0.18 |
| **Memory Dual** | **0.76*** | | | **1** | | 0.11 | 0.19 | 0.51* | 0.36* | -0.13 | -0.1 | -0.33* |
| **Processing Single** | **0.28*** | | | **0.30*** | | **1** | 0.62* | 0.07 | 0.07 | -0.24* | -0.36* | 0.13 |
| **Processing Dual** | **0.23*** | | | **0.23*** | | **0.76*** | **1** | 0.18 | 0.07 | -0.25* | -0.38* | -0.53* |
| **Running Span** | **0.46*** | | | **0.42*** | | **0.20*** | **0.12** | **1** | 0.22* | -0.17 | -0.08 | -0.11 |
| **Silly Sentences** | **0.33*** | | | **0.35*** | | **0.24*** | **0.18** | **0.23*** | **1** | -0.1 | 0.03 | -0.04 |
| **Simple RT** | **-0.18** | | | **-0.11** | | **-0.41*** | **-0.38*** | **-0.13** | **-0.03** | **1** | 0.43* | 0.11 |
| **Choice RT** | **-0.24*** | | | **-0.25*** | | **-0.61*** | **-0.52*** | **-0.23*** | **-0.15** | **0.41*** | **1** | 0.12 |
| **Dual-task cost** | **0.15** | | | **-0.35*** | | **0.05** | **-0.39*** | **0.04** | **-0.04** | **-0.03** | **0.05** | **1** |
| B. Correlations in all participants (*N* = 539) | | | | | | | | | | | | |
|  | | **Memory Single** | | | **Memory Dual** | **Processing Single** | **Processing Dual** | **Running Span** | **Silly Sentences** | **Simple RT** | **Choice RT** |  |
| **Memory Single** | | | 1 | |  |  |  |  |  |  |  |  |
| **Memory Dual** | | | 0.77* | | 1 |  |  |  |  |  |  |  |
| **Processing Single** | | | 0.33* | | 0.39* | 1 |  |  |  |  |  |  |
| **Processing Dual** | | | 0.30* | | 0.37* | 0.83* | 1 |  |  |  |  |  |
| **Running Span** | | | 0.50* | | 0.48* | 0.24* | 0.22* | 1 |  |  |  |  |
| **Silly Sentences** | | | 0.32* | | 0.32* | 0.12 | 0.09 | 0.22* | 1 |  |  |  |
| **Simple RT** | | | -0.19* | | -0.18* | -0.41* | -0.39* | -0.18* | -0.05 | 1 |  |  |
| **Choice RT** | | | -0.29* | | -0.36* | -0.71* | -0.66* | -0.25* | -0.06 | 0.45* | 1 |  |
| **Dual-task cost** | | | 0.09 | | -0.39* | -0.1 | -0.49* | -0.07 | -0.03 | 0.08 | 0.19* |  |

*Note. ** Represents significant correlations at *p* < .001.

| *Table S5.* Correlations between odd and even number trials (and split-half reliability) in all participants (*N* = 539) | | | | | | | | | | |
| --- | --- | --- | --- | --- | --- | --- | --- | --- | --- | --- |
|  | **Memory Single** | | **Memory Dual** | **Processing Single** | **Processing Dual** | **Running Span** | **Silly Sentences** | **Simple RT** | **Choice RT** |  |
| **Memory Single** | | .36* (.53) |  |  |  |  |  |  |  |  |
| **Memory Dual** | |  | .40* (.57) |  |  |  |  |  |  |  |
| **Processing Single** | |  |  | .71* (.83) |  |  |  |  |  |  |
| **Processing Dual** | |  |  |  | .70*(.83) |  |  |  |  |  |
| **Running Span** | |  |  |  |  | .24* (.39) |  |  |  |  |
| **Silly Sentences** | |  |  |  |  |  | - |  |  |  |
| **Simple RT** | |  |  |  |  |  |  | .11** (.20) |  |  |
| **Choice RT** | |  |  |  |  |  |  |  | 0.38* (.55) |  |
|  | |  |  |  |  |  |  |  |  |  |

*Note. ** Represents significant correlations at *p* < .001, and ** Represents significant correlations at *p* < .05. Due to the adaptive nature (i.e., task difficulty increased based on performance, or the task ended) of the Silly Sentences task, calculating split-half reliability for this measure was not appropriate. For the split-half reliability (values in parentheses), we used the Spearman-Brown Prophecy formula, to correct for the correlation being for only half of the trials, which systematically reduces the magnitude of the correlation.

*Table S6.* Task performance correlations in all participants, corrected for attenuation (*N* = 539)

|  |  |  |  |  |  |  |  |  |  |  |
| --- | --- | --- | --- | --- | --- | --- | --- | --- | --- | --- |
|  | | **Memory Single** | | **Memory Dual** | **Processing Single** | **Processing Dual** | **Running Span** | **Silly Sentences** | **Simple RT** | **Choice RT** |
| **Memory Single** | | |  | 1.0 | 0.50 | 0.45 | 1.0 | - | -0.58 | -0.54 |
| **Memory Dual** | | |  |  | 0.57 | 0.54 | 1.0 | - | -0.53 | -0.64 |
| **Processing Single** | | |  |  |  | 1.0 | 0.42 | - | -1.0 | -1.0 |
| **Processing Dual** | | |  |  |  |  | 0.39 | - | -0.96 | -0.98 |
| **Running Span** | | |  |  |  |  |  | - | -0.64 | -0.54 |
| **Silly Sentences** | | |  |  |  |  |  |  | - | - |
| **Simple RT** | | |  |  |  |  |  |  |  | 1.0 |
|  | | |  |  |  |  |  |  |  |  |
|  | | |  |  |  |  |  |  |  |  |
|  | | |  |  |  |  |  |  |  |  |

*Note.* Correlations between task performance on the different tasks were corrected for attenuation, to take into account the fact that when measures are less reliable, there is a limit to how much they can correlate with each other. These values are an estimation of what the correlation would be if the measures were perfectly reliable. It is calculated by dividing the original correlation between the two measures, by the square root of the two task reliabilities (see Table S5), multiplied by each other. Here, this correction was implemented using the ‘disattenuated.cor’ function in the R ‘CTT’ package. Disattenuated values greater than 1.00 indicate that measurement error is not randomly distributed. We report all disattenuated correlations greater than 1.0 as 1.0. Finally, due to the adaptive nature (i.e., task difficulty increased based on performance, or the task ended) of the Silly Sentences task, calculating split-half reliability for this measure was not appropriate.

*Table S7.* Average Response Times (RTs) across trials in the Processing Tasks (*N* = 539)

|  | **Single** | | | **Dual** | |  |
| --- | --- | --- | --- | --- | --- | --- |
| **Item order** | **RT (ms)** | | **N** | **RT (ms)** | **N** |  |
| 1 | | 949.6 | 5386 | 1744.8 | 5367 |  |
| **2** | | 752.3 | 5385 | 892.9 | 5350 |  |
| **3** | | 744.0 | 5363 | 802.1 | 5283 |  |
| **4** | | 730.2 | 5310 | 755.4 | 5122 |  |
| **5** | | 719.0 | 5174 | 698.0 | 4666 |  |
| **6** | | 688.0 | 4814 | 643.4 | 3974 |  |
| **7** | | 646.0 | 4109 | 587.6 | 3041 |  |
| **8** | | 581.6 | 2957 | 526.1 | 2045 |  |
| **9** | | 517.3 | 1662 | 473.3 | 1104 |  |
| **10** | | 438.0 | 503 | 418.9 | 331 |  |
| **11** | | 355.0 | 52 | 359.2 | 25 |  |
| **12** | | 187.8 | 4 | 33 | 1 |  |
| **13** | | 150.5 | 2 |  |  |  |
| **14** | | 122 | 1 |  |  |  |
| **15** | | 114 | 1 |  |  |  |
| **16** | | 134 | 1 |  |  |  |

*Note.* N represents the number of processing task responses at this specific item order, for each task type.

*Table S8.* Average Response Times across trials in the Silly Sentences Task (*N* = 539)

| **Number of Memory Items** | **Response Time (ms)** | **N** |  |
| --- | --- | --- | --- |
| 2 | 3717.3 | 1078 |  |
| 3 | 4061.5 | 934 |  |
| 4 | 5105.0 | 730 |  |
| 5 | 6113.7 | 568 |  |
| 6 | 7339.1 | 382 |  |
| 7 | 9494.7 | 212 |  |

*Table S9*. Participant language status by age group (*N* = 539)

| **Age Range** | ***N*** | **Monolingual English speaker from birth** | **Bilingual/Multilingual including English speaker from birth** | **Not a native English speaker - learned English at school or later in life (now use English in my daily life)** | **Not a native English speaker - learned English at school or later in life (do not use English in my daily life)** |
| --- | --- | --- | --- | --- | --- |
| **15–24** | 132 | 58 (43.9%) | 26 (19.7%) | 47 (35.6%) | 1 (0.8%) |
| **25–34** | 64 | 27 (42.2%) | 4 (6.3%) | 21 (32.8%) | 12 (18.8%) |
| **35–44** | 37 | 14 (37.8%) | 4 (10.8%) | 16 (43.2%) | 3 (8.1%) |
| **45–54** | 49 | 39 (79.6%) | 3 (6.1%) | 6 (12.2%) | 1 (2.0%) |
| **55–64** | 86 | 73 (84.9%) | 9 (10.5%) | 4 (4.7%) | 0 (0.0%) |
| **65–74** | 129 | 115 (89.1%) | 9 (7.0%) | 3 (2.3%) | 2 (1.6%) |
| **75–90** | 42 | 41 (97.6%) | 1 (2.4 %) | 0 (0.0%) | 0 (0.0%) |

*Table S10.* Bayes Factor evidence participants in a given age group performed differently from *all older participants* in the study (i.e., all participants older than the participants in the specified age group), in only monolingual, English-speaking participants (*N* = 367).

| **Age group** | **Memory Single** | **Processing Single** | **Running Span** | **Silly**  **Sentences** | **Simple RT** | **Complex RT** | **Memory Dual-Task** **(relative/proportional)** | **Processing Dual-Task (relative/proportional)** | **General Dual-Task**  **(relative/proportional)** |
| --- | --- | --- | --- | --- | --- | --- | --- | --- | --- |
| 15 – 24 | *0.16* | **9.1 × 10^17^** | 0.30 | *0.41* | **98.2** | **4.7 × 10^12^** | **68.2 /** **29.1** | **5.1 /** **3.8** | **164.1/** **597.2** |
| 25 – 34 | **6.8** | **1.5 × 10^11^** | **4.10** | 0.27 | *0.73* | **2.5 × 10^6^** | *0.91 / 1.25* | **27.7/** *1.0* | *2.2 /* **5.5** |
| 35 – 44 | *1.4* | **1.8 × 10^3^** | *0.86* | 0.30 | 0.28 | *2.22* | *0.45 / 0.38* | 0.19 / *0.35* | *0.42 / 0.48* |
| 45 – 54 | *0.34* | **2.0 × 10^4^** | 0.19 | *0.66* | **3.9** | **1.1 × 10^3^** | 0.19 / 0.24 | 0.18 / *0.60* | 0.32 / *0.53* |
| 55 – 64 | 0.24 | **79.7** | 0.25 | *0.43* | *0.78* | *2.3* | 0.18/ 0.16 | 0.15 / 0.28 | 0.16 / 0.18 |
| 65 – 74 | *0.65* | **17.2** | *0.39* | *2.9* | **6.6** | *0.45* | 0.29 / *0.36* | 0.29 / 0.23 | 0.20 / 0.23 |

*Note.* Values in **bold** represent evidence in favor of an age difference, *italics* represent inconclusive evidence, and underscored represents evidence against an age difference. The *Dual-Task* measures represent the standardised relative dual-task costs (i.e., how much performance declined between the single and dual-task versions of a given task; values to the left, and proportional differences between the single and dual-task versions; values to the right).

*Table S11*. Average task performance by age group, in only monolingual, English-speaking participants (*N* = 367).

| **Age Group** | **Memory**  **Single (accuracy)** | **Memory Dual (accuracy)** | **Processing Single (correct responses)** | **Processing Dual (correct responses)** | **Running Span (accuracy)** | | **Silly**  **Sentences (maximum level reached)** | **Simple**  **RT (in ms)** | **Choice**  **RT (for correct trials; in ms)** |
| --- | --- | --- | --- | --- | --- | --- | --- | --- | --- |
| **15 - 24** | 0.68 (0.15) | 0.70 (0.17) | 8.3 (0.7) | 7.7 (1.2) | 0.50 (0.09) | 4.4 (1.7) | | 291 (45) | 427 (89) |
| **25 - 34** | 0.77 (0.17) | 0.77 (0.15) | 8.3 (0.7) | 7.7 (1.4) | 0.52 (0.06) | 4.9 (1.9) | | 303 (58) | 440 (58) |
| **35 - 44** | 0.76 (0.18) | 0.75 (0.22) | 7.8 (1.0) | 7.0 (1.1) | 0.52 (0.09) | 4.5 (1.5) | | 320 (60) | 540 (94) |
| **45 - 54** | 0.69 (0.20) | 0.64 (0.21) | 7.2 (0.9) | 6.5 (1.2) | 0.48 (0.11) | 5.1 (1.7) | | 301 (36) | 536 (130) |
| **55 - 64** | 0.67 (0.19) | 0.61 (0.23) | 6.5 (1.1) | 5.7 (1.4) | 0.48 (0.11) | 4.9 (1.5) | | 315 (45) | 621 (161) |
| **65 - 74** | 0.66 (0.20) | 0.60 (0.22) | 6.1 (1.2) | 5.2 (1.3) | 0.47 (0.09) | 4.7 (1.7) | | 322 (49) | 667 (175) |
| **75 - 90** | 0.60 (0.19) | 0.56 (0.22) | 5.4 (1.1) | 4.4 (1.4) | 0.45 (0.10) | 4.0 (1.6) | | 354 (91) | 709 (148) |

*Note*. Mean values by age group (only for monolingual, Native English speakers), values in parenthesis represent the standard deviation.
